# Solvent-accessibility of discrete residue positions in the polypeptide hormone glucagon by ^19^F-NMR observation of 4-fluorophenylalanine

**DOI:** 10.1007/s10858-017-0107-8

**Published:** 2017-05-15

**Authors:** Yaguang Hou, Wanhui Hu, Xiaona Li, John J. Skinner, Dongsheng Liu, Kurt Wüthrich

**Affiliations:** 1 iHuman Institute, ShanghaiTech University, 393 Middle Huaxia Road, Shanghai, 201210 China; 2 Institute of Biochemistry and Cell Biology, Shanghai Institutes for Biological Sciences, Chinese Academy of Sciences, 320 Yue Yang Road, Shanghai, 200031 China; 3 University of the Chinese Academy of Sciences, Beijing, 100049 China; 4 State Key Laboratory of Drug Research, Shanghai Institute of Materia Medica, Chinese Academy of Sciences, Shanghai, 201203 China; 5 Department of Integrative Structural and Computational Biology and the Skaggs Institute for Chemical Biology, The Scripps Research Institute, La Jolla, CA 92037 USA; 6 School of Life Science and Technology, ShanghaiTech University, 393 Middle Huaxia Road, Shanghai, 201210 China

**Keywords:** ^19^F-NMR, Glucagon, ^19^F chemical shift, Solvent exposure from D_2_O effects

## Abstract

The amino acid 4-fluoro-l-phenylalanine (4F-Phe) was introduced at the positions of Phe6 and Phe22 in the 29-residue polypeptide hormone glucagon by expressing glucagon in *E. coli* in the presence of an excess of 4F-Phe. Glucagon regulates blood glucose homeostasis by interaction with the glucagon receptor (GCGR), a class B GPCR. By referencing to the 4F-Phe chemical shifts at varying D_2_O concentrations, the solvent exposure of the two Phe sites along the glucagon sequence was determined, showing that 4F-Phe6 was fully solvent exposed and 4F-Phe22 was only partially exposed. The incorporation of fluorine atoms in polypeptide hormones paves the way for novel studies of their interactions with membrane-spanning receptors, specifically by differentiating between effects on the solvent accessibility, the line shapes, and the chemical shifts from interactions with lipids, detergents and proteins. Studies of interactions of GCGR with ligands in solution is at this point of keen interest, given that recent crystallographic studies revealed that an apparent small molecule antagonist actually binds as an allosteric effector at a distance of ~20 Å from the orthosteric ligand binding site (Jazayeri et al., in Nature 533:274–277, 2016).

## Introduction

This paper describes work toward the mapping of interactions between the polypeptide hormone glucagon and the glucagon receptor (GCGR), which is a Class B G protein-coupled receptor (GPCR) (Hollenstein et al. 2014). Polypeptides can adopt a much wider range of conformations than the small-molecule drugs bound in most GPCR crystal structures (e.g., Chrencik et al. 2015; Hua et al. 2016; Jazayeri et al. 2016). Nuclear magnetic resonance (NMR) spectroscopy is a powerful tool for determining polypeptide conformations (e.g., Wüthrich 2003), but, due to line broadening and signal overlap, much of the NMR information is typically lost upon binding of the polypeptides to larger molecules. We plan to overcome these limitations by using ^19^F-NMR probes, which have chemical shifts that are well separated from those of the macromolecular background (Didenko et al. 2013). The ^19^F van der Waals radius of 1.35 Å is close to that of hydrogen (1.20 Å), and ^19^F is widely considered as being isosteric with ^1^H (Marsh and Suzuki 2014). High sensitivity to detection and large chemical shift dispersion have enabled ^19^F-NMR studies of protein folding/unfolding, intermolecular interactions and conformational dynamics (Chen et al. 2013; Didenko et al. 2013; Kitevski-LeBlanc and Prosser 2012). ^19^F-NMR studies of GPCRs have so far mainly been based on post-translational chemical conjugation with cysteine sulfhydryl groups, for example, with TET (2,2,2-trifluoroethanethiol) (Klein-Seetharaman et al. 1999; Liu et al. 2012) or BTFA (3-bromo-1,1,1-trifluoroacetone) (Kim et al. 2013). Application of this approach for the labeling of polypeptide ligands changes the chemical structure much more extensively than the incorporation of ^19^F-containing amino acids. We therefore use the latter strategy in this work based on supplying *E. coli* cells with an excess of 4F-Phe, which was then incorporated *via* the naturally occurring tRNA^Phe^.

Glucagon is a 29-amino acid polypeptide hormone processed from the precursor proglucagon in pancreatic α-cells (Jiang and Zhang 2003). Glucagon plays a crucial role in the regulation of blood glucose homeostasis by stimulating glycogenolysis and gluconeogenesis in hepatocytes (Mayo et al. 2003) and lipolysis in adipocytes (Perea et al. 1995). Glucagon functions by binding to and thus activating GCGR, which is one of the 15 members of the secretin-like (class B) family of human GPCRs (Hollenstein et al. 2014; Siu et al. 2013). Binding of glucagon to GCGR results in activation of intracellular G proteins and adenylate cyclase, and the resulting increase in intracellular cyclic adenosine monophosphate (cAMP) levels initiates the next steps in the signaling pathway (Jiang and Zhang 2003; Rodgers 2012).

**Fig. 1:**
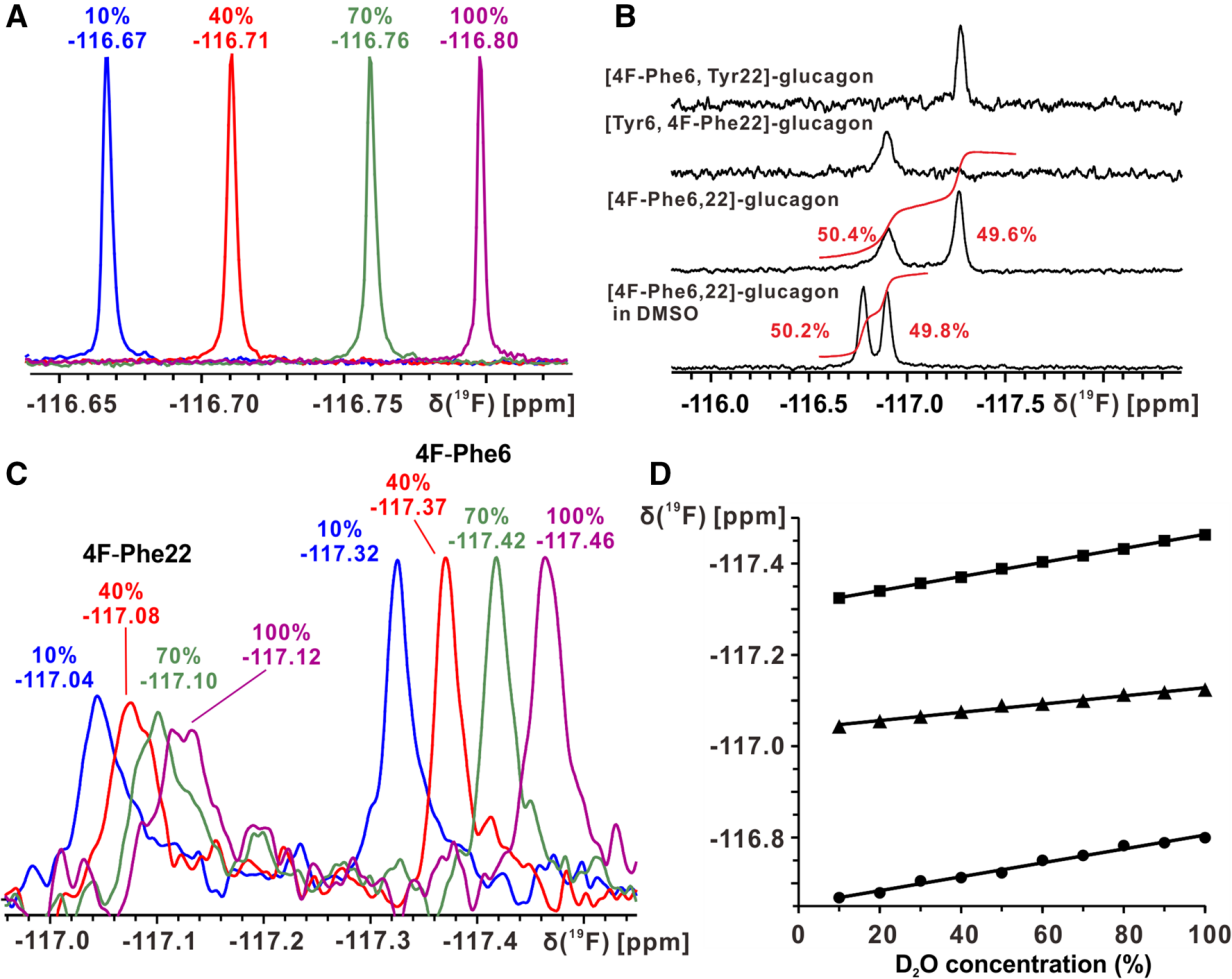
1D ^19^F-NMR spectra at 470 and 565 MHz of three glucagon variants and of 4F-Phe. **a** Four 470 MHz spectra of 4 mM 4F-Phe in water containing, respectively, 10, 40, 70 and 100% D_2_O. The percentage of D_2_O and the measured chemical shift are shown *above* each spectrum. **b** 565 MHz spectra of 0.3 mM solutions of 4F-Phe-labeled glucagon variants in H_2_O solution containing 10% D_2_O; the doubly-labeled glucagon was also measured in DMSO solution. For the two [4F-Phe6,22]-glucagon spectra, the integration traces and relative peak intensities are shown in *red*. These spectra were recorded with a Bruker Avance-600 spectrometer equipped with a cryogenic TCI probehead; no ^1^H-decoupling was applied during ^19^F-detection. **c** Four spectra of 0.1 mM [4F-Phe6,22]-glucagon in water containing, respectively, 10, 40, 70 and 100% D_2_O, as shown at the *top* of the peaks, where the measured chemical shifts are also indicated. **d** *Plots versus* the D_2_O concentration of the ^19^F chemical shifts measured in (**a**) and (**c**) for 4F-Phe (*filled circle*), and for 4F-Phe6 (*filled square*) and 4F-Phe22 (*filled triangle*) in [4F-Phe6,22]-glucagon

**Fig. 2:**
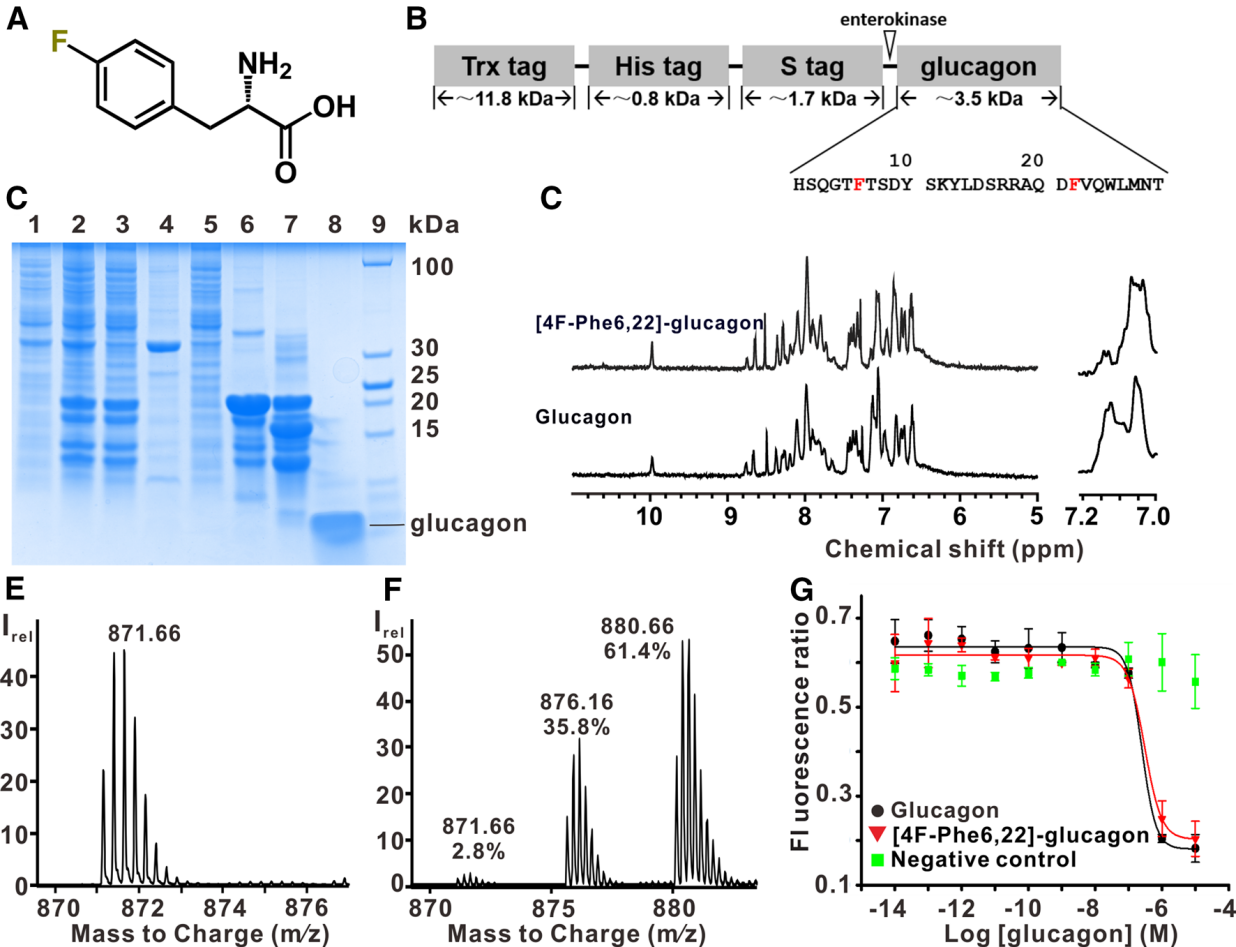
Chemical structure and biophysical/biochemical characterization of [4F-Phe6,22]-glucagon. **a** Chemical structure of 4-fluoro-l-phenylalanine (4F-Phe). **b** Scheme of the Trx-His-S-glucagon fusion protein used for glucagon expression, where a Trx tag, a 6 × His tag, an S tag, and an enterokinase cleavage site (DDDDK↓) precede glucagon. In the amino acid sequence of glucagon, Phe6 and Phe22 are highlighted in *red*. **c** SDS–PAGE gel documenting the expression and purification of glucagon. *Lane 1* crude extract of uninduced *E. coli* BL21 cells (DE3); *lane 2* cell extract at 5 h after induction; *lanes 3* and *4* supernatant and pellet, respectively, after sonication; *lane 5* flow-through from the Co^2+^-TALON column; *lane 6* Co^2+^-TALON elution with 250 mM imidazole; *lane 7* same as 6 after enterokinase treatment; *lane 8* same as 7 after HPLC and lyophilization; *lane 9* molecular weight markers. **d** 1D ^1^H-NMR spectra of [4F-Phe6,22]-glucagon and wild type glucagon. The spectral region from 10 to 5 ppm is shown, with 7.2 to 7.0 ppm enlarged on the *right*. **e** Mass spectrometry of glucagon. **f** Mass spectrometry of the [4F-Phe6.22]-glucagon preparation used for the ^19^F-NMR measurements. In **e** and **f**, the peaks represent the molecules with four positive charges by additional protons, and the m/z ratios of the highest peaks of these peak clusters are indicated. In **f**, an estimate of the relative abundance of unlabeled, singly-labeled and doubly-labeled glucagon is indicated below the m/z ratio of each peak, respectively. **g** cAMP assays. cAMP formation was monitored by the fluorescence ratio 665/615 nm. The *error bars* represent the standard error of the mean. A sigmoidal fit of the data is shown. The EC_50_ values for glucagon and [4F-Phe6,22]-glucagon were 0.249 and 0.304 μM, respectively

Here, glucagon was labeled by substituting Phe in positions 6 and 22 with the non-proteinogenic amino acid 4-fluoro-l-phenylalanine (4F-Phe) (Bann and Frieden 2004; Li and Frieden 2005). The solvent isotope effect on the ^19^F chemical shift at variable D_2_O concentrations (Hansen et al. 1985; Kitevski-LeBlanc et al. 2009; Shi et al. 2011) was used to investigate site-specific solvent accessibility of glucagon (Fig. 1). As a control, 1D ^1^H-NMR and cAMP assays were used to show that 4F-Phe incorporation caused at most minimal perturbations of glucagon conformation and activity (Fig. 2). Our work lays a foundation for studying glucagon conformation and dynamics when interacting with GCGR with and without the presence of intramolecular partner proteins. Further investigation of glucagon–GCGR interactions in solution is particularly important given recent crystallographic evidence that an apparent small-molecule antagonist actually binds as an allosteric effector to the intracellular side of GCGR at the distance of ~20 Å from the orthosteric ligand binding site (Jazayeri et al. 2016).

### ^19^F-NMR study of glucagon solvent accessibility at discrete sequence locations

The two phenylalanine residues in positions 6 and 22 of the glucagon amino acid sequence were substituted by 4F-Phe (Fig. 2a) in the recombinant variant polypeptide (Fig. 2b) by incubating *E. coli* cells with an excess of 4F-Phe, which was then incorporated *via* the naturally occurring tRNA^Phe. 19^F-NMR spectra were obtained for 4F-Phe and for 4F-Phe-labeled glucagon in H_2_O/D_2_O solutions with variable D_2_O concentrations (Fig. 1a, c). Samples of 4 mM 4F-Phe and 0.1 mM 4F-Phe-labeled glucagon in acidic water (pH^*^ 2.5) were prepared with addition of D_2_O at 10% increments for ^19^F-NMR spectra collection. Within the NMR tube, a capillary containing 5 mM trifluoroacetic acid (TFA) was used as an external standard, and ^19^F chemical shift values were referenced to TFA at −76.55 ppm. Except for the spectra shown in Fig. 1b (see the figure caption for details), the ^19^F-NMR experiments were performed at 298 K on a Bruker AVANCE-500 spectrometer equipped with a room temperature BBO probehead (Bruker Biospin), using the pulse program zgfhigqn30.2 with ^1^H-decoupling during ^19^F-detection. The ^19^F frequency was about 470 MHz, and the number of scans was 128 for 4F-Phe, and 2048 for 4F-Phe-labeled glucagon. Prior to Fourier transformation, the time-domain NMR data were multiplied with an exponential function with line broadening factors of 1 and 5 Hz for 4F-Phe and 4F-Phe-labeled glucagon, respectively.

The ^19^F-NMR signals for all the samples shifted upfield with increasing D_2_O/H_2_O ratio. For 4F-Phe, the ^19^F chemical shift difference between 10 and 100% D_2_O was about 0.14 ppm (Fig. 1a). This is consistent with previous reports on other systems (Hansen et al. 1985; Kitevski-LeBlanc et al. 2009; Shi et al. 2011).

For [4F-Phe6,22]-glucagon, the ^19^F-NMR spectrum showed two peaks, representing the two fluorine-labeled sites (Fig. 1b), which had been introduced by incubating *E. coli* cells with an excess of 4F-Phe. In order to assign the two peaks, two glucagon mutants were designed, as detailed in Fig. 1b. The ^19^F-NMR spectra of each of these two mutants showed only one resonance, with the 4F-Phe6 signal upfield from the 4F-Phe22 signal (Fig. 1b).

In the ^19^F-NMR spectrum, 4F-Phe6 had a significantly narrower resonance peak than 4F-Phe22. For 4F-Phe6, the full widths at half peak height were about 13, 13, 14 and 15 Hz at the D_2_O concentrations of 10, 40, 70 and 100%, respectively, and for 4F-Phe22 the corresponding values were about 20, 23, 24 and 23 Hz, respectively (Fig. 1c).

Just as with 4F-Phe, the ^19^F chemical shift values of both labels in glucagon decreased linearly with increasing D_2_O concentration (Fig. 1d). The maximum ^19^F chemical shift differences were 0.14 ppm for 4F-Phe6 and 0.08 ppm for 4F-Phe22. These differences suggest that position 6 has full solvent exposure, whereas position 22 is only partially exposed to solvent. This result is readily rationalized by structural data from previous ^1^H-NMR studies of glucagon in acidic aqueous solutions, which showed that the region around residue six is flexibly disordered and Phe22 is part of a hydrophobic cluster formed by the residues –Phe22–Val23–Gln24–Trp25– (Boesch et al. 1978).

### Glucagon conformation and biological activity are preserved after 4F-Phe incorporation

The glucagon fusion construct used for expression in *E. coli* contained an N-terminal Trx (thioredoxin) tag, a His tag, an S tag, and an enterokinase cleavage site (LaVallie et al. 1993) (Fig. 2a, b). For expression we used an M9 culture medium. 0.5 g/L of 4F-Phe was added when the OD_600_ reached 0.6. After 30 min, protein expression was induced with 0.5 mM IPTG, and cells were subsequently grown overnight at 25 °C. We did not notice a possible toxic effect of 4F-Phe at the aforementioned concentration. After purification with a Co^2+^-TALON superflow affinity column (Clontech), the proteins were incubated with enterokinase (BBI Life Science) to cleave the N-terminal Trx tag, His tag, and S tag. Expression and purification of glucagon were analyzed by SDS/PAGE (Fig. 2c). The yield of purified glucagon was 3 mg from 1 L of *E. coli* culture.

Glucagon has poor solubility in aqueous buffers at physiological pH. Therefore, Trx fusion was used as a solubility enhancement tag to express glucagon (Chabenne et al. 2010). The N-terminal histidine residue of glucagon has an important role in modulating the GCGR signaling pathway (Sueiras-Diaz et al. 1984). Here, we therefore used enterokinase to cleave the Trx-His-S-glucagon fusion protein; this protease cleaves after lysine, resulting in no additional residues at the glucagon N-terminus, as shown by mass spectroscopy (Fig. 2e, f).

The incorporation of 4F-Phe into glucagon was analyzed using liquid chromatography electro-spray ionization mass spectrometry (LCESI-MS) (Fig. 2e, f). For unlabeled glucagon, the peaks were consistent with the theoretical molecular weight (3482.5 Da). For [4F-Phe6,22]-glucagon, because of variable extents of 4F-Phe incorporation, there were three peak clusters with molecular weights of 3482.50, 3500.61 and 3518.59 Da, which were assigned to unlabeled, singly and doubly 4F-Phe-labeled glucagon, respectively, each carrying 4 H^+^. According to the relative peak intensities, we estimated that the relative abundance of unlabeled, singly-labeled and doubly-labeled glucagon were about 3, 36, and 61%, respectively.

1D ^1^H-NMR was applied to detect the influence of ^19^F-labeling on the conformation of glucagon. Samples of [4F-Phe6,22]-glucagon and wild type glucagon in 400 µL of acidic water containing 10% D_2_O (pH^*^ 2.5) were prepared, and the ^1^H-NMR experiments were performed at 298 K on the aforementioned Bruker AVANCE-500 spectrometer. The ^1^H-NMR spectra of [4F-Phe6,22]-glucagon and wild type glucagon were almost identical; some differences in the spectral region from 7.0 to 7.2 ppm are likely due to effects from the substitution of H^ζ^ by ^19^F on the other Phe ring hydrogens (Fig. 2d).

Glucagon activates adenylate cyclase by binding to GCGR and activating G proteins (Bataille and Dalle 2014). We applied cAMP assays (Norskov-Lauritsen et al. 2014) to test [4F-Phe6,22]-glucagon for activity. The binding curves demonstrated that [4F-Phe6,22]-glucagon had similar activity as glucagon (Fig. 2g), indicating that this ^19^F-labeling does not affect glucagon signaling activity.

## Discussion

In the present paper we used recombinant technology to replace Phe in glucagon with the nonproteinogenic amino acid 4F-Phe by incubating *E. coli* cells with an excess of 4F-Phe, since replacement by 4F-Phe causes minimal perturbation of the covalent structure. As hoped for, this substitution had at most minimal effects on the structure and the biological activity of glucagon. The same approach could of course be used for the introduction of more attractive ^19^F-labels such as, for example, CF_3_-Phe (Chen et al. 2013).

Mass-spectrometric (MS) analysis indicated that about 80% of Phe was replaced by 4F-Phe with the experimental conditions used. Combined with the ^19^F-NMR spectra in Fig. 1b, we can further conclude that the two Phe-positions in glucagon are nearly equally occupied by 4F-Phe in [4F-Phe6,22]-glucagon. The MS data (Fig. 2f) also showed that the procedure used yielded a mixture of singly and doubly 4F-Phe-labeled glucagon. However, since the chemical shifts and line shapes of the two labeling sites are identical in singly and doubly labeled glucagon (Fig. 1b), interaction studies with GCGR could be performed with either one of the three constructs in Fig. 1b, or with a mixture of singly and doubly labeled glucagon as obtained here.

It is reassuring to note that the observations on solvent accessibility and line shapes for 4F-Phe6 and 4F-Phe22 can be rationalized with a previous NMR structure determination of glucagon in acidic aqueous solution, which showed that Phe6 is in a flexibly disordered region of the polypeptide, while Phe22 is part of a hydrophobic cluster (Boesch et al. 1978). The chemical shift difference between 4F-Phe6 and 4F-Phe22 of 0.28 ppm, which enables to use both labeling sites for independent observations in a single experiment, reflects the locally different conformations along the glucagon chain. This is clearly documented by comparison of the ^19^F-NMR spectra of [4F-Phe6,22]-glucagon in H_2_O and DMSO (Fig. 1b); it has been shown previously that DMSO reversibly unfolds the hydrophobic cluster formed by the glucagon residues 22–25 (Boesch et al. 1978; Bundi et al. 1978). It will be interesting to investigate interactions of [4Phe6,22]-glucagon with GCGR not only on the basis of solvent accessibility and line shape variations due to decreased mobility of the bound glucagon and possibly conformational microheterogeneity, but also on the basis of the chemical shifts, which may provide information on conformational changes of the glucagon upon binding. Further investigation of glucagon binding to GCGR in solution will be critical for exploring of the role of the newly discovered allosteric site located at the distance of ~20 Å from the orthosteric ligand binding site of GCGR (Jazayeri et al. 2016).
